# An Artificial Functional Family Filter in Homolog Searching in Next-generation Sequencing Metagenomics

**DOI:** 10.1371/journal.pone.0058669

**Published:** 2013-03-14

**Authors:** Ruofei Du, Donald Mercante, Zhide Fang

**Affiliations:** Biostatistics Program, School of Public Health, Louisiana State University Health Sciences Center, New Orleans, Louisiana, United States of America; University of Westminster, United Kingdom

## Abstract

In functional metagenomics, BLAST homology search is a common method to classify metagenomic reads into protein/domain sequence families such as Clusters of Orthologous Groups of proteins (COGs) in order to quantify the abundance of each COG in the community. The resulting functional profile of the community is then used in downstream analysis to correlate the change in abundance to environmental perturbation, clinical variation, and so on. However, the short read length coupled with next-generation sequencing technologies poses a barrier in this approach, essentially because similarity significance cannot be discerned by searching with short reads. Consequently, artificial functional families are produced, in which those with a large number of reads assigned decreases the accuracy of functional profile dramatically. There is no method available to address this problem. We intended to fill this gap in this paper. We revealed that BLAST similarity scores of homologues for short reads from COG protein members coding sequences are distributed differently from the scores of those derived elsewhere. We showed that, by choosing an appropriate score cut-off, we are able to filter out most artificial families and simultaneously to preserve sufficient information in order to build the functional profile. We also showed that, by incorporated application of BLAST and RPS-BLAST, some artificial families with large read counts can be further identified after the score cutoff filtration. Evaluated on three experimental metagenomic datasets with different coverages, we found that the proposed method is robust against read coverage and consistently outperforms the other E-value cutoff methods currently used in literatures.

## Introduction

Microbes play important roles in ecosystems and human health. Human microbes, such as bacteria, virus, and fungi, are either beneficial or harmful to health, depending on the conditions. In order to understand microbial function and adaptation, it has become very important to accurately quantify the abundance of each functional feature within different conditions, and to reveal the association between the change in abundance and the environmental/clinical perturbation.

Metagenomics, the study of genomic material extracted directly from environmental samples [1], is different from traditional methodologies in that it does not rely on isolation and cultivation of single microbe species. Its use promises new insight into microbial community structural and functional properties. Depending on sequencing technology, metagenomic studies can be divided into Sanger Shotgun sequencing and next-generation sequencing (NGS) metagenomic approaches. A disadvantage of Sanger sequencing is its susceptibility to possible cloning bias [2]. Using cloning-free based NGS technologies can eliminate the possibility of this bias, and thus, are more suitable to metagenomic studies. Furthermore, in contrast to the traditional chain-termination methods, NGS technologies utilize a more efficient, array-based work flow to determine the nucleotide base order of a DNA fragment. These differences make NGS technologies more desirable for conducting metagenomic studies than the traditional technology in at least two important aspects: speed and cost [3]. NGS metagenomic sequencing reads contain information regarding the gene content of a microbial community. Protein coding sequences within genes can reveal potential biochemical functions of the community. Based on sequence similarity, a BLAST homolog search tool is used to classify metagenomic reads into COG protein/domain families whose functions have been well annotated [4], [5]. The functional profile of a community is then represented by the proportions of reads being classified into associated COG families. This is the so-called read count approach [6].

However, the lengths of the NGS reads may pose a barrier to this approach. Specifically, non-homologous sequences can share regional sequence similarities, which can introduce artificial functional families when aligning the short reads. By artificial functional families (or artificial COGs) we mean those COG families whose member proteins do not exist in the metagenome. The mechanism of artificial COGs being generated can be explained by a simple (but realistic) example. Suppose there is a DNA non-coding sequence *A* of length 1000 nt. If the intact sequence *A* is aligned against the COG database by BLAST, there will not be any homolog with statistical significance. Assume that there is a sequence of consecutive 80 bases in *A* which can be aligned to a sequence in COG *a*. When *A* is fragmented into short reads averaging 100 nt long and aligned against the COG database, there may be one read that can be detected homologous to COG *a* with statistical significance. This is a false homolog regarding functional annotation of the read(s), and COG *a* will appear in the profile with at least one read count. If, in fact, the metagenome does not have any function associated to COG *a*, this COG is an artificial one. In current approaches, the BLAST E-value is usually used to discern the significance of sequence similarity. Various ‘conventional’ cutoff E-values have been used, such as 10^−3^ [7], [8], 10^−5^ [9], or 10^−1^∼10^−8^ [10]. Setting too loose a cutoff for selecting homologues introduces too much noise, and setting too strict a cutoff will result in missing many true homologues [8]. In simulation studies conducted by our group, we found that the BLAST similarity scores of homologues for short reads derived from COG protein member coding sequences (COG-CDS) regions and from elsewhere are distributed differently. The difference motivated us to derive a better score cutoff value, instead of a ‘conventional’ E-value cutoff.

We used a simulated data set to compare alignments generated by BLAST and Reserved Position Specific BLAST (RPS-BLAST) respectively. BLAST and RPS-BLAST alignments usually assign different read counts to the same COG. Considering the COGs with large read counts (defined later), we observed that the ratio of read counts (RRC) by RPS-BLAST and the BLAST is distributed differently for the artificial COGs as compared to other COGs. Thus, we used this ratio as the classification feature to further filter influential artificial families (defined later).

Additionally, experimentally simulated metagenome data sets were used to evaluate the performances of our method and two current methods in the literature. Our method outperforms the others in keeping the fidelity of the functional profile.

## Materials and Methods

### The COG database

Sequence homology designates a relationship of sharing common ancestry [11]. Orthologs are defined as homologous genes in different species that originated from a common ancestral sequence [12]. The COG database was constructed based on this definition, towards the purpose of phylogenetic classification of the proteins encoded in different microbial genomes. Basically, each family in the COG database was formed by measuring sequence similarity with the BLAST tool, such that the protein members in a family are more homologous to one another than they are to other proteins from the same genome [13]. Since orthologous proteins generally perform the same biochemical function in the cell, a common practice in functional genomics and recently emerged metagenomics is to use the functional information of the well-characterized proteins to annotate the orthologous proteins that are newly discovered or less studied [4], [14], [15].

The NCBI COG database, derived from 66 microbial genomes, consists of 4873 COG families. These families are classified into 25 categories, such that COG families have more similar functional character within a category than between categories [16].

### BLAST and RPS-BLAST

BLAST was developed to search locally similar sequences (DNA or amino acid) with gaps allowed, and to compute the significance of similarity. Due to its efficient computing algorithm and theoretical rationalization, BLAST has become a favorable tool for sequence similarity alignment [17], [18]. In this study, we used BLASTX, a member in BLAST tool box (version 2.2.25+), to find proteins similar to translated DNA sequences.

In BLAST, sequence similarity for a local alignment is quantified by its aggregate score, computed by adding substitution scores for aligned pairs of letters, and subtracting penalty scores for gap openings and gap extensions. A local alignment, whose aggregate score cannot be improved by local extension or trimming, is called a high-scoring segment pair (HSP). The aggregate score is denoted as the Maximal Segment Score (MSS) [18]. For the comparison of two random sequences of lengths 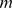
and 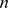, the asymptotical distribution of the MSS is well studied when the alignment is ungapped, that is, when no insertions and deletions exist. Under an appropriate scoring system, when 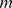 and 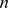
are sufficiently large, we can model the distribution of MSS by an extreme value distribution, that is,
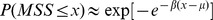
where both the scale parameter 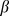 and the location parameter
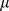
depend on 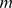, 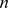, and the scoring matrix [19], [20]. The E-value is calculated as the expected number of distinct local HSPs with MSS being at least 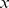
and in practice is used frequently to evaluate the significance of similarity [20], [21]. When gaps are considered in alignment, simulation studies further suggest that, with appropriate gap open and extension penalties, the distribution remains true [18]. In this case, the parameters can be estimated [22].

One assumption in BLAST is that amino acids at different positions are independent. This assumption is needed to derive the distribution of MSS, but it results in the BLAST's failure to consider the patterns of conservation in homologues. Position Specific BLAST (PSI-BLAST) was then developed to recognize the pattern embodied in homologues, and to use the pattern instead of the simple query to BLAST against protein sequences in the database [18]. PSI-BLAST, unlike BLAST, has shown the ability to identify distant relatives of a protein. RPS-BLAST, in contrast, searches a query against the patterns recognized from the database, as its name indicates [23]. In this paper, we used RPSTBLASTN, one member program in RPS-BLAST (version 2.2.25+).

### Simulation of 454 Reads from Reference Genome Sequences

We randomly selected 10 bacteria genomes to simulate short reads to demonstrate our method. **Table 1** lists the brief information about these genomes. The reference genome sequences were downloaded from the NCBI ftp site. The site also contains the annotation files of COG-CDS locations on the genomes.

**Table 1:** The list of genomes for the simulation of short reads.

Genome Accession #	Organism Name	Genome Length (nt.)	No. CDSs	No. COG-CDSs
NC_007778	*Rhodopseudomonas palustris* HaA2	5331656	4683	3666
NC_008255	*Cytophaga hutchinsonii* ATCC 33406	4433218	3785	2327
NC_007644	*Moorella thermoacetica* ATCC 39073	2628784	2463	2003
NC_007354	*Ehrlichia canis Jake*	1315030	925	678
NC_007404	*Thiobacillus denitrificans* ATCC	2909809	2827	2231
NC_007335	*Prochlorococcus sp.* NATL2A	1842899	2162	1197
NC_007925	*Rhodopseudomonas palustris* BisB18	5513844	4886	3708
NC_007947	*Methylobacillus flagellatus strain* KT	2971517	2753	2231
NC_007406	*Nitrobacter winogradskyi* Nb-255	3402093	3122	2351
NC_007958	*Rhodopseudomonas palustris* BisB5	4892717	4397	3400

We used a sequencing simulator, MetaSim (version 0.9.5) [24], to generate the sequencing reads. This simulator takes into account the sequencing error and has options of different technology platforms (we chose 454 sequencing reads in this paper). The average read length was set to be 100 nt. For simplicity, the sequence coverage was set to be 1. We showed later that, with experimental data sets, our approach is robust against different coverages (<1,  = 1, and >1). The pool of all these reads made up the simulated metagenome data. The numbers of simulated reads, corresponding to the genomes in **Table 1**, are 53368, 44469, 26104, 13300, 29226, 18457, 55135, 29825, 33705 and 48826, respectively.

### Empirical Distributions of Similarity Scores of TSHs and FSHs

Generally, more than one homolog will be found when a short read is aligned against a database of sequences. Among them, a homology is termed Best-hit Homolog (BH) if it has the most significant similarity ― the largest similarity score. It is a common practice to associate a short read to its BH in functional annotations of metagenomes [5], [10], [25]. We adopted this strategy for the simulated reads, and if multiple homologues shared the largest similarity score, we randomly picked one. Hereafter, we will refer to short read, BH, and short homology interchangeably for convenience.

**Figure 1:**
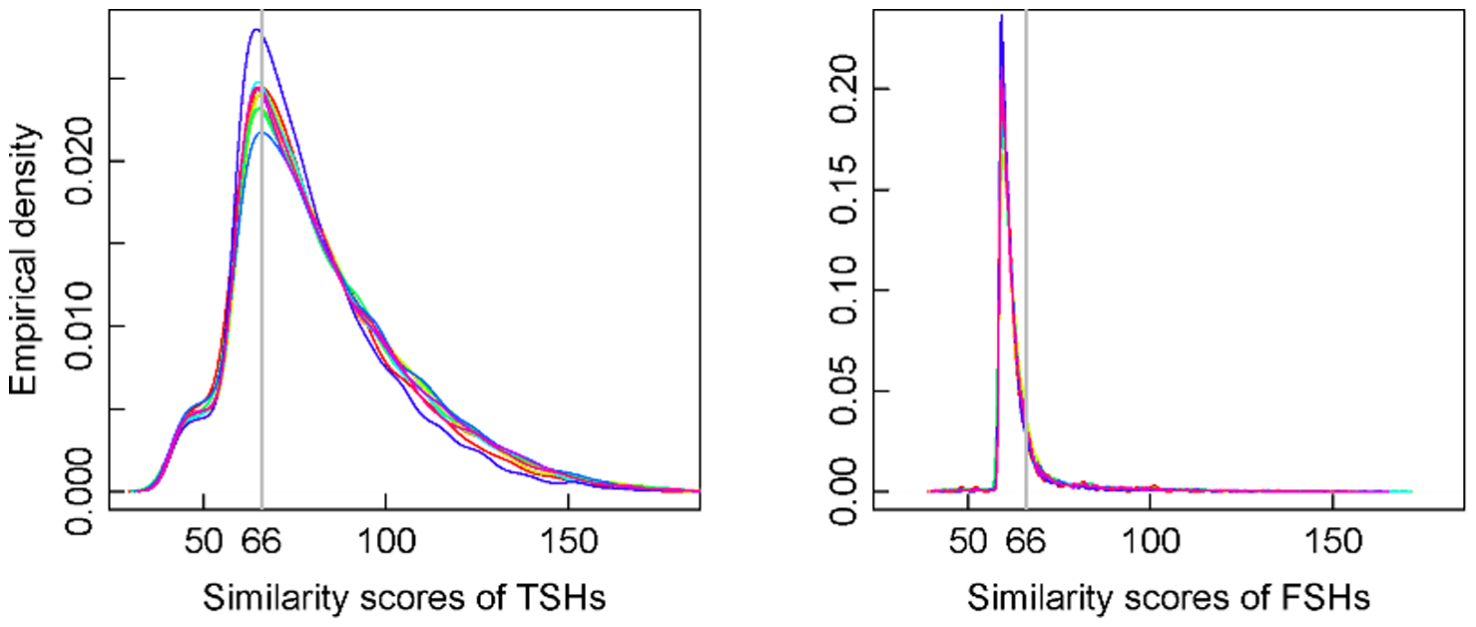
Empirical densities of similarity scores for TSHs and FSHs. The curves with the same color represents, for each of the 10 genomes, the smoothed empirical densities of similarity scores of TSHs (left panel) and FSHs (right panel) separately. The vertical grey lines demonstrate the score cutoff of 66.

In the simulation, the genome locations of the short reads are known. We expect that if the corresponding genome location overlaps with a COG-CDS region by at least 60 nt (the same criterion used in [6]), a short read can be aligned to a sequence in the COG family. If the aligned BH homologous sequence does belong to the expected COG, the alignment is marked as True Short Homology (TSH); otherwise False Short Homology (FSH). We obtained the empirical distributions of similarity scores of TSHs and FSHs across the 10 genomes. In **Figure 1**, we present the smoothed empirical densities for overlaying similarity scores of TSHs (left panel) and FSHs (right panel) separately, with one color representing one of the 10 genomes.

We have the following observations from **Figure 1**. (1) the empirical densities of the similarity scores of TSHs have the same pattern across genomes, so are those of FSHs. (2) The distributions for TSHs are bimodal, with the lower modes being close to 45 and the upper modes close to 66. For FSHs, the distributions are unimodal, with the peaks being close to 59. (3) The similarity scores of FSHs tend to be smaller (<66), while those of TSHs tend to be larger (>66). The sample means, around the score value of 63, of the FSH scores are smaller than those, around 80, of the TSH scores. For each genome, one-sample T-tests imply that the mean score is either significantly less than 66 (FSH) or significantly greater than 66 (TSH). Furthermore, the third sample quantiles of the FSH scores are less than 65, while all the first sample quantiles of the TSH scores are already close to 65. All these suggest that TSHs are more likely to cluster in the higher score region, while FSHs tend to cluster in the lower score region.

### Filtration by a similarity score cutoff

**Figure 2:**
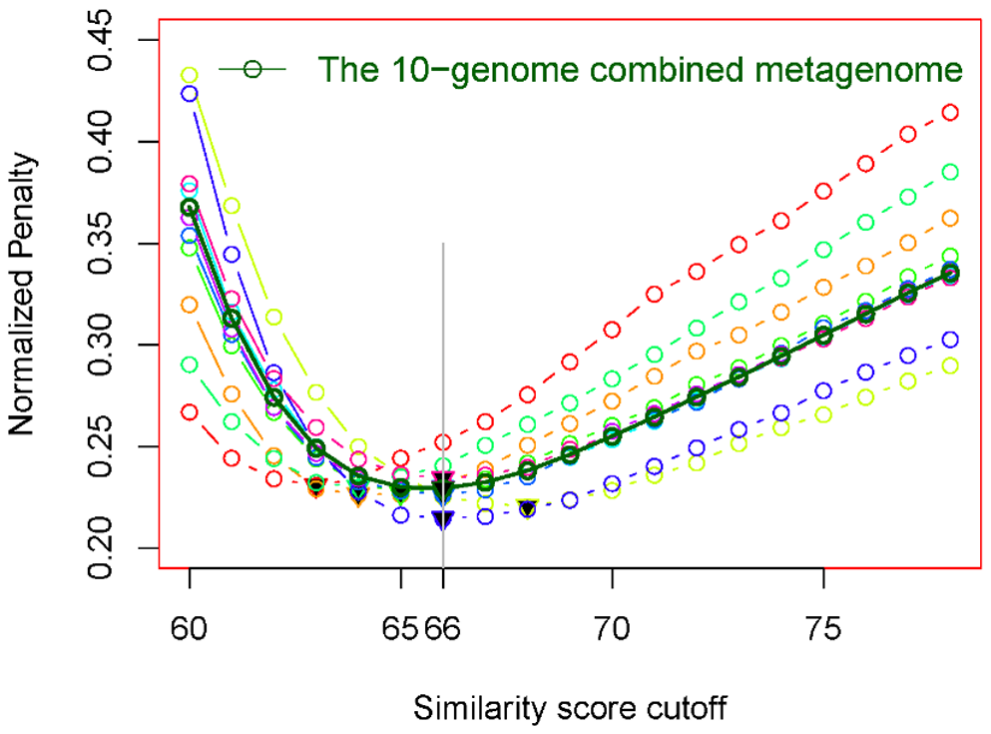
The plot of the normalized penalty versus the score cutoff value. The bold dark green curve is for the simulated combined metagenome, and the other colored curves are for single genomes. On each curve, the filled black point-down triangle denotes the least normalized penalty.

The above observations motivated us to find a ‘good’ cutoff score in order to classify the BH homologues into TSHs and FSHs. We used the 0–1 penalty rule for this purpose. The rule puts penalty 0 for a correct classification and penalty 1 for a misclassification. Then the ratio of the total penalties and the number of homologues to be classified is defined as the normalized penalty. We plot normalized penalty versus score cutoff value (from 60 to 78) in **Figure 2**, with the bold dark green curve being for the simulated 10-genome combined metagenome. It is clear that the cutoff score of 66 has the lowest normalized penalty for the metagenomic data. We also present in this figure normalized penalty curves for single genomes in the metagenome (each colored curve, except the bold dark green one, represents one genome). On each curve, a filled black point-down triangle denotes the least normalized penalty. These triangles consistently show that the score cutoff value of 66 results in the (approximately) lowest normalized penalty, and thus can serve as a ‘good’ cutoff for BLAST output of the short reads with about 100 nt bases.

### Sensitivity of the similarity score cutoff at homolog level

By a score cutoff, we classify a BH as TSH if its similarity score being greater or equal to the cutoff value; otherwise, it is marked as FSH. Then, the sensitivity at homolog level is defined as the proportion of TSHs correctly classified. Applying similarity score cutoff of 66 in the simulated ∼100 nt metagenome, the achieved sensitivity is 0.74 (66545/89859). We demonstrate, later in this paper that, based on these reserved TSHs we are able to construct a more accurate functional profile than those by the two E-value cutoff methods in literatures.

We comment that in practice, the composition proportions of similarity scores from genomes may differ, and thus the score cutoff to reach the least penalty may be different from 66. We show in Supporting Information (**Text S1**) that, to achieve a sensitivity of about 0.75, we are almost sure (probability>99%) that the score cutoff value should be in the range of 63 to 68, regardless of what the composition proportions are. From the fact that the 10 genomes were selected randomly, we can assume that the other genomes have the similar pattern in the distributions of the similarity scores of TSHs. Thus, the conclusion about the score cutoff can be generalized to any NGS ∼100 nt short read metagenome.

### Sensitivity at COG level and the trivial loss of true COG families by the similarity score cutoff

The process of classifying the BHs by a similarity score cutoff results in the classification of the associated COGs into true COGs and artificial COGs. A COG is classified as an artificial COG if all of its BHs have similarity scores less than the score cutoff, otherwise, it is classified as a true COG. In this subsection, we will first report sensitivity and specificity of the classification at aggregate (COG) level, which are the proportions of true COGs preserved and artificial COGs being filtered out separately. The rational for this investigation is because ultimately the functional profile is presented by the collection of the count of BHs in each COG. Furthermore, we studied the loss of true COG families and concluded they are trivial entries in the entire functional profile.

There are 3040 true COGs in the simulated metagenome. BLAST detected a total of 4310 COGs, out of which there are 2959 true COGs and 1351 artificial COGs (see **Table 2**). While BLAST is a valid tool for metagenomic functional analysis in successfully identifying 97% (2959/3040) of the true COG families, it introduced large number of artificial COGs (31%). This justifies the need of filtration to the BLAST output.

**Table 2:** Numbers of True COGs with numbers of TSHs covered and numbers of artificial COGs, before and after filtration by score cutoff.

	True COGs		Artificial COGs
	No. of COGs	No. of TSHs covered	No. of COGs
Raw BLAST output	2959	89859	1351
Filtered BLAST output by Score cutoff of 66	2702	89747	312

By using the score cutoff value of 66 and classifying a BH homologue as TSH if its similarity score is greater or equal to 66, we successfully filtered out 1039, out of 1351 artificial COGs, which indicate a decent specificity of 77% (1039/1351). The classification resulted in a loss of 257 true COGs (**Table 2**) from those identified by BLAST, but still maintains a sensitivity of 91.3% (1–257/2959). Furthermore, for any of these lost true COGs, there are no BHs assigned with similarity score being 66 or larger, even though this cutoff value is low and around the 25% quantile of the empirical distribution of scores of TSHs for a genome. This implies that sequences within any of these lost COGs do not share high sequence conservations. On the other hand, there are in total 112 TSHs covered by these lost true COGs - averaging 0.4 TSH in one lost true COG - and we found the largest number of TSHs in one lost true COG is only 4. These are trivial entries for the functional profile, because in a profile there are usually many (>100) other COGs and each of them has more than 100 TSHs assigned.

### Incorporation of Results by BLAST and RPS-BLAST to Further Identify Influential artificial COGs

Generally, though filtration by score cutoff can filter out most of the artificial COGs, it may miss some, especially those with large counts (termed influential artificial COGs next). By incorporating the alignment outputs of BLAST and RPS-BLAST, we applied the quadratic discriminant analysis (QDA) to identify and remove such influential artificial COGs. Next, we will explain why RPS-BLAST output is needed and demonstrate that QDA is effective.

In the case of perfect alignment, if no or very few coding sequences relate to a COG, there should be no or very few short reads being aligned to the COG. However, as mentioned above, BLAST can erroneously annotate a short read fragmented from a non-coding sequence to a COG function. This can result in a read count for an artificial COG, or a large read count for a COG with trivial abundance. One reason is that BLAST does not consider conservation pattern embodied within true homologies. RPS-BLAST, in contrast, considers such pattern. Thus, RPS-BLAST may be more sensitive to exclude FSHs, and then assign smaller read count to an artificial COG or a COG with trivial abundance. The difference in read counts assigned to a COG by RPS-BLAST and BLAST can then be used to identify artificial COG.

**Figure 3:**
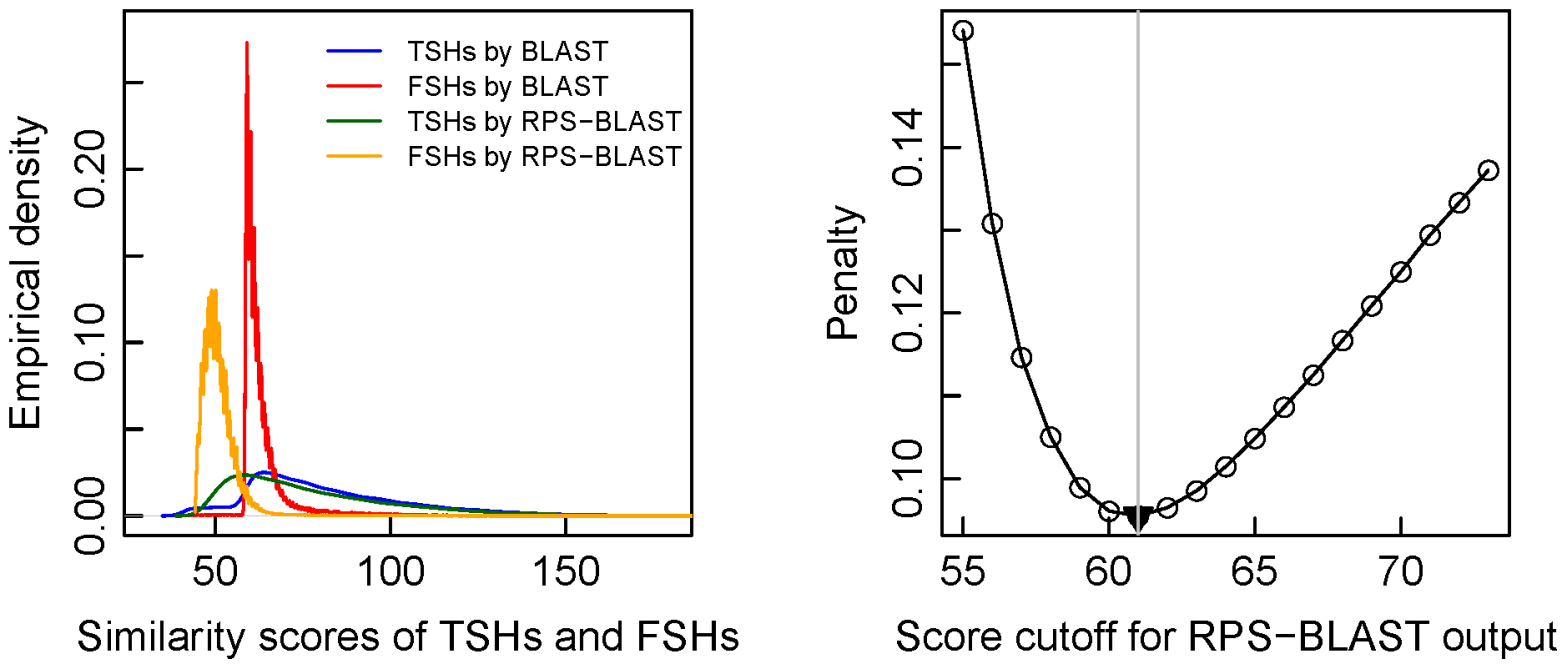
Empirical densities of similarity scores by BLAST and RPS-BLAST (left) and the normalized penalty plot by RPS-BLAST (right). The plot demonstrates that the penalty is minimized at the similarity score of 61.

In other aspect, RPS-BLAST is good at finding more distant homologues. The simulated reads were aligned against COG database by RPS-BLAST (the specific tool is RPSTBLASTN). As above, a read was annotated by its BH. RPS-BLAST detected 184,381 more annotated reads than BLAST did. However, the results generated by BLAST and RPS-BLAST show a noticeable difference in the proportions of TSHs, with 61% (89859 TSHs in total 148305 annotated reads) by BLAST and 25% (83435 TSHs in total 332686 annotated reads) by RPS-BLAST. The proportion drop from 61% to 25% attests that RPS-BLAST inflates false homologues. This is a less favorable feature of RPS-BLAST. Nevertheless, RPS-BLAST remains competitive because FSHs from its output are more clustered in the lower-score region, and thus are easy to be filtered out. This characteristic is demonstrated in **Figure 3** (the left panel) for the simulated ∼100 nt metagenome. The related normalized penalty curve (the right panel of **Figure 3**) is minimized at the similarity score of 61 (the least penalty is 0.095). Though the least penalty is less than that (0.23 in **Figure 2**) given by using BLAST output, the RPS-BLAST produces more artificial COGs than BLAST. For example, for the simulated ∼100 nt metagenome, there are 789 false COGs in RPS-BLAST output being filtered at cutoff score 61, and 312 false COGs in BLAST output being filtered at cutoff score 66. Thus, our proposed strategy is to first filter out false COGs in the BLAST-generated functional profile, and then use the profile from RPS-BLAST output as a supporting tool to further remove those remaining influential artificial COGs (defined next). By so doing, we can take advantage of RPS-BLAST for keeping out FSHs which do not share the same conservation pattern, and at the same time avoiding the negative impact of RPS-BLAST.

Before defining the influential artificial COG, we extend the definition of artificial COG for this stage. That is, in addition to those without any protein in the metagenome, a COG with a very small proportion of classified reads from TSHs is considered as an artificial COG too. An artificial COG with a large read count will skew a functional profile dramatically. We call such COG an influential artificial COG. Our goal is to identify and remove these entries. We do not give explicit quantities for ‘very small proportion’ and ‘large read count’ here, but recommend that, after filtration by score cutoff, one may examine the COGs with read counts above 95th percentile and above 75th percentile by the following **Step 1** and **Step 2**.

**Step 1**. We first define the influential artificial COGs and then describe the method in this step to detect them. After the score filtration, we obtained a class of COGs with 95% percentile or above read counts. If one of such COGs has the proportion of the reads from TSHs being less than 0.1, it is defined as an influential artificial COG. By this definition, there are five influential artificial COGs in the simulated ∼100 nt metagenome data (**Table 3**).

**Table 3:** Influential artificial COGs defined in the simulated ∼100 nt metagenome.

COGs	No. of CDSs in the metagenome	BLAST result (after score filtration)		
		Read Count assigned	Read Count from TSHs	Proportion of reads from TSHs
COG0457	1	93	2	0.02
COG0477	0	447	0	0
COG0500	0	193	0	0
COG2202	8	223	15	0.07
COG3210	1	110	7	0.06

**Figure 4:**
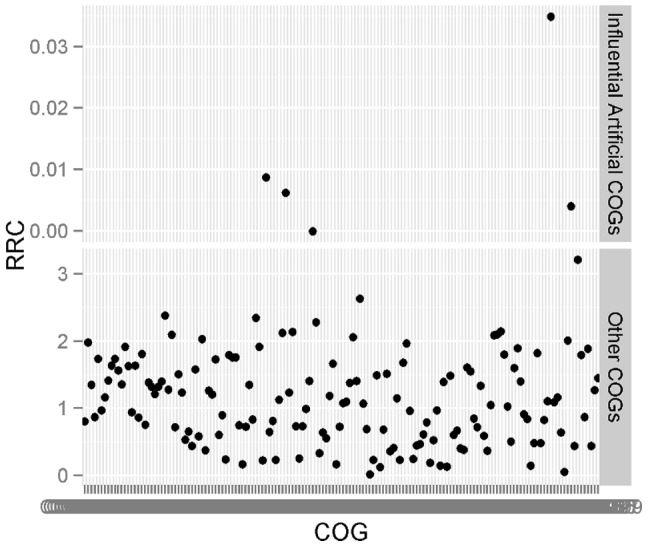
The plot of RRCs for the COGs with 95% percentile or above read counts. The RRC values for five influential artificial COGs range between 0 and 0.04. For the other COG families, the RRCs are farther away from 0, with only one RRC being less than 0.05.

To detect these influential artificial COGs, it is interesting to observe that, among all the COGs with 95% percentile or higher read counts, the RRCs, calculated by read counts before score filtration, for influential artificial COGs (**Table 3**) are quite different from those for other COGs (**Figure 4**). Specifically, among the COGs with large read counts, the five influential artificial COGs have RRCs ranging from 0 to 0.04, while the RRCs for other COG families are farther away from 0, with only one RRC less than 0.05. Thus the RRC can be used as the feature for the classification of COGs.

We applied QDA, one of the popular nonlinear approaches for classification, to detect influential artificial COGs. Let 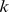 be the index of 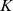 groups, 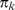 denote the probability of class 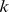
with 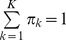, 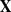 be the vector of all features and 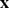 as its observed value. Suppose that the probability density 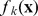 of 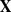 for the 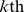 group is a multivariate Gaussian with mean vector 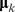 and variance-covariance matrix 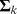. Then a general QDA function can be written as [26],
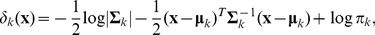
(1)


where 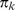, 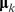, and 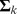 are estimated from a training data set. The classification for a future 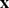 can be determined by comparing the value of 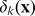.

Using the simulated metagenomic data as the training data set, with RRC as its sole feature, we obtained maximum likelihood estimates: 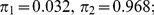 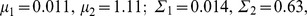 where subscript 1 and 2 refer to the influential artificial and true COG groups respectively. Our proposed classifier is calculated by plugging these values in equation (1). Four influential artificial COGs, COG0477, COG0500, COG3210 and COG0457, were identified by applying our method to the training dataset.

**Step 2**. There may be some influential artificial COGs whose read counts are smaller than 95th percentile and thus cannot be identified by **Step 1**. This step focuses on detecting such COGs. After BLAST score filtration, for the class of COGs with 75% percentile or above read counts, we proposed that, if a COG family has zero read count in RPS-BLAST profile after score filtration (similarity score >61), it is classified as an influential artificial COG. The rationale of this step is that a true COG should have reads assigned by both BLAST and RPS-BLAST. By this rule, two COGs were further identified (**Table 4**), of which COG0454 was not detected in **Step 1**.

**Table 4:** Influential artificial COGs identified in **Step 2**.

COG	No. of CDSs in metagenome	Read Count by BLAST	Read Count by BLAST after score filtration	Read Count by RPS-BLAST after score filtration
COG0454	0	159	34	0
COG0500	0	511	193	0

The detection results in both steps, together with those in the next section, indicate that the classifier is successful. We comment that using QDA, over linear discriminant analysis, is because of the inequality of two variances. There is no evidently violation of normality of RRCs in the true COG group because the p value of normality test is relative large (p value is 0.04 in Kolmogorov-Smirno test, or 0.03 in Cramer-von Mises test) and the boxplot (not shown) is very symmetric. Note that a small number of influential artificial COGs make it difficult to check the normality assumption in this group. It is possible that other more complicated classifier such as kernel quadratic discriminant analysis performs better than QDA. The evaluation of different classifiers is interesting and will be our future research topic.

### The work flow of the proposed method

**Figure 5:**
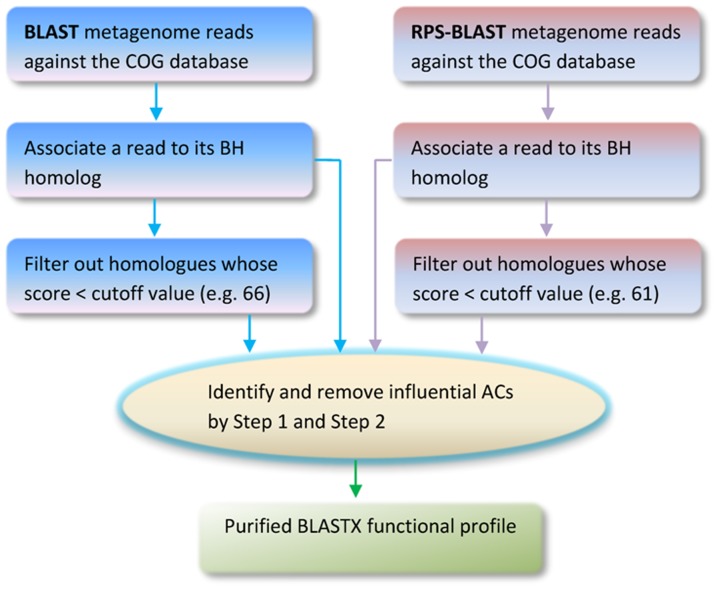
Work-flow for the proposed method.

The proposed method can be summarized in the following work flow chart (**Figure 5**).

## Results

In this section, we report the effect of our proposed method on the accuracy of the functional profiles for both the simulated metagenome and experimental metagenomes with different coverages.

### Improved functional profile on the simulated metagenome

**Figure 6:**
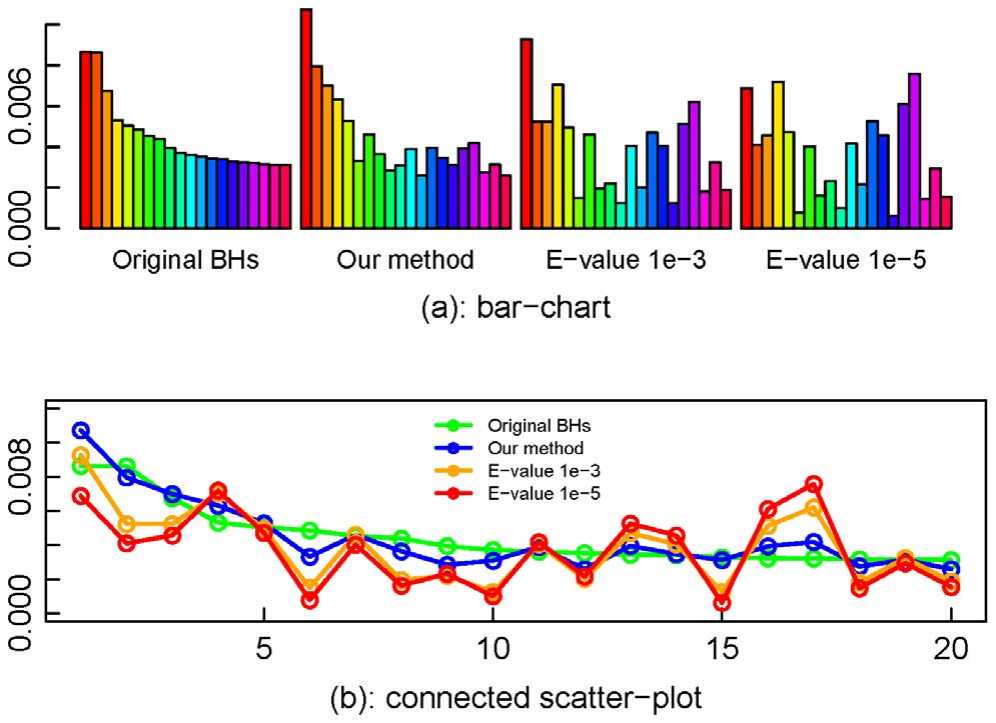
Partial functional profiles for the simulated metagenome by different methods. The plots indicate that, comparing with the E-value cutoff methods, the proposed method produces a function profile most similar to that in the original BHs.

We constructed and compared the partial functional profiles, using original BLAST BHs, our proposed method, two current methods in the literatures (that is, homologues which have E-values less than 10^−3^ or 10^−5^ were remained) separately. Only included are true COGs in the functional profile built by the original BLAST BHs. Without loss of generality, we selected for comparison the top 20 COGs with the highest proportions in the profile by the original BLAST BHs for comparison. The corresponding proportions in the functional profiles built by the other three methods are plotted in **Figure 6** (each bar in the plots of the top panel represents the proportion of one COG). In terms of the similarity to the partial profile in the original BHs, our proposed method outperforms those using E-value cutoffs. Note that for complete profiles, the sums of absolute differences of proportions between the original BHs and our proposed method, E-value method with cutoff 10^−3^, and E-value method with cutoff 10^−5^ are 0.21, 0.43 and 0.53, respectively. This implies that using the homologues with very small E-values alone to generate functional profiles not only decreases the sensitivity of true COGs but also affects the proportion of abundance for a COG in the profile. In other words, the read count for a COG collected solely from regions with very large similarity scores is not enough to represent the read count abundance with fidelity.

### Effect of the read coverage

The read coverage is usually defined as the ratio of the total length of all short reads sequenced and the total number of the bases in the metagenome. It varies from one sample to another. We used three metagenome data sets in [10], named M3_01X, M3_1X, and M3_2X, as the benchmark data sets to evaluate the performance of the proposed method at different read coverages. In the file names, ‘01X’, ‘1X’ and ‘2X’ denote the read coverages of 0.1, 1 and 2 respectively. The numbers of reads in these data sets are 35865, 353054, and 706113, respectively. Each data set consists of the same 8 genomes, sequenced on the 454 GS20 platform to produce short reads of ∼100 nt at the Joint Genome Institute, US Department of Energy. Different from the simulated dataset used above, these data were generated through a genuine sequencing platform. Thus the read length distribution and the sequencing errors may differ from simulations. Since we know which genomes are included in the dataset, we are able to evaluate the performance of the methods.

A brief summary of these eight genomes is given in **Table 5**. We obtained a total 3153 distinct families of COG-CDSs on these genomes, based on NCBI genome protein annotations.

**Table 5:** Information of genomes in the data sets.

GenomeAccession NO.	Organism Name	GenomeLength (nt.)	No. of CDSs	No. ofCOG-CDSs
NC_009438	*Shewanella putrefaciens* CN-32	4659200	3972	3125
NC_009092	*Shewanella loihica* PV-4	4602594	3859	3050
NC_008789	*Halorhodospira halophila* SL1	2678452	2407	1987
NC_009512	*Pseudomonas putida* F1	5959964	5250	4202
NC_009997	*Shewanella baltica* OS195	5347283	4499	3391
NC_011593	*Bifidobacterium longum bv. Infantis* ATCC 15697	2832748	2416	1656
NC_011071	*Stenotrophomonas maltophilia* R551-3	4573969	4039	3092
NC_009719	*Parvibaculum lavamentivorans* DS-1	3914745	3636	2943

### Results from the data set with read coverage less than 1

Analysis of the dataset M3_01X detected 2836 COGs using BLAST to search and associate a read to its BH homolog. Of them, 2502 were true COGs and 334 artificial COGs. After applying a score cutoff of 66, we successfully reduced the number of artificial COGs from 334 to 64 (i.e. 81% successfully filtered out). Note that applying the score cutoff resulted in the loss of 313 true COGs (12.5%), averaging 1.5 reads per COG with a maximum of 8 reads per COG. The loss of these COGs has negligible impact on the overall construction of the functional profile. Furthermore, by applying the trained QDA function (**Step 1**), we detected three influential artificial COGs (**Table 6**). The small estimated numbers of existing CDSs in the table indicate that the detection was successful.

**Table 6:** Influential artificial COGs in M3_01X detected by Step 1 and Step 2.

	COGs	Estimated No. of truly existing CDSs	Read Counts	
			By BLAST	By RPS-BLAST
Step 1	COG0454	0	35	0
	COG0500	0.2	54	0
	COG2202	0.3	62	2
Step 2	COG0457	0	15	0
	COG1226	0.5	7	0
	COG2214	0	8	0
	COG3210	0	7	0

Note: Two columns of read counts are obtained before score filtration (Step 1) and after score filtration (Step 2).

Applying **Step 2** resulted in another four influential artificial COGs successfully identified (**Table 6**). These results indicate that the combination of proposed methods work effectively for the metagenomic data with read coverage less than 1.

**Figure 7:**
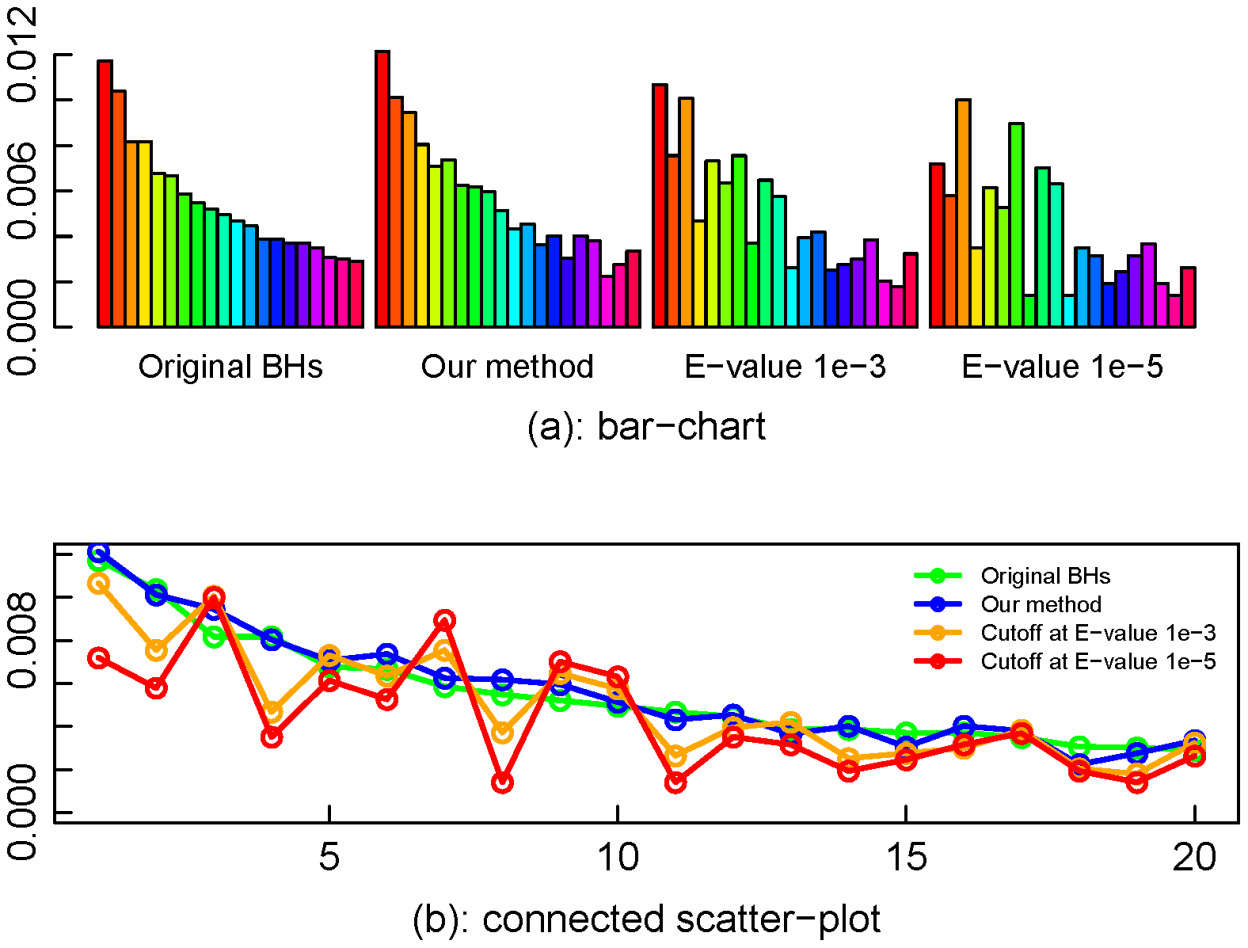
Partial functional profiles for M3_01X by different methods. The plots show that for the data set with coverage less than 1, the proposed method performs better than the two E-value cutoff methods in keeping the fidelity of the functional profile.

**Figure 7**, similar to **Figure 6**, shows the partial functional profiles with the 20 largest abundant COGs, using our proposed method and the two E-value cutoff methods. The plots imply that our method achieves the best profile fidelity for the metagenomic data with read coverage less than 1. For the complete profiles, the sums of absolute differences of proportions between the original BHs and our proposed method, E-value method with cutoff 10^−3^, E-value method with cutoff 10^−5^ and are 0.19, 0.32 and 0.41 respectively.

### Results from the data set with read coverage equal to 1

BLAST searching of dataset M3_1X detected 4285 COGs, including 3108 true COGs and 1177 artificial COGs. Again, BLAST introduced a large proportion (27%) of artificial COGs. By applying the score cutoff of 66, we filtered out 76% of these COGs, resulting in 284 artificial COGs. Consequently, we lost 154 (5%) true COGs, each averaging 3.3 short reads with the maximum read count of 20. Again, these have negligible impact in the functional profile. Additional influential artificial COGs were identified by Steps 1 and 2 (**Table 7**). Note that the values of RRC for the COGs in **Step 1** are 0, 0, and 0.037, respectively. In conclusion, our proposed method is successful in identifying and filtering out the false COGs and influential artificial COGs for the metagenomic data with read coverage equal to 1.

**Table 7:** Influential artificial COGs in M3_1X detected by Step 1 and Step 2.

	COG ID	Estimated No. of truly existing CDSs	Read Counts	
			By BLAST	By RPS-BLAST
Step 1	COG0454	0	362	0
	COG0500	2	658	0
	COG0477	0	2283	85
Step 2	COG0457	0	101	0
	COG0639	2	58	0

Note: Two columns of read counts are obtained before score filtration (Step 1) and after score filtration (Step 2).

**Figure 8:**
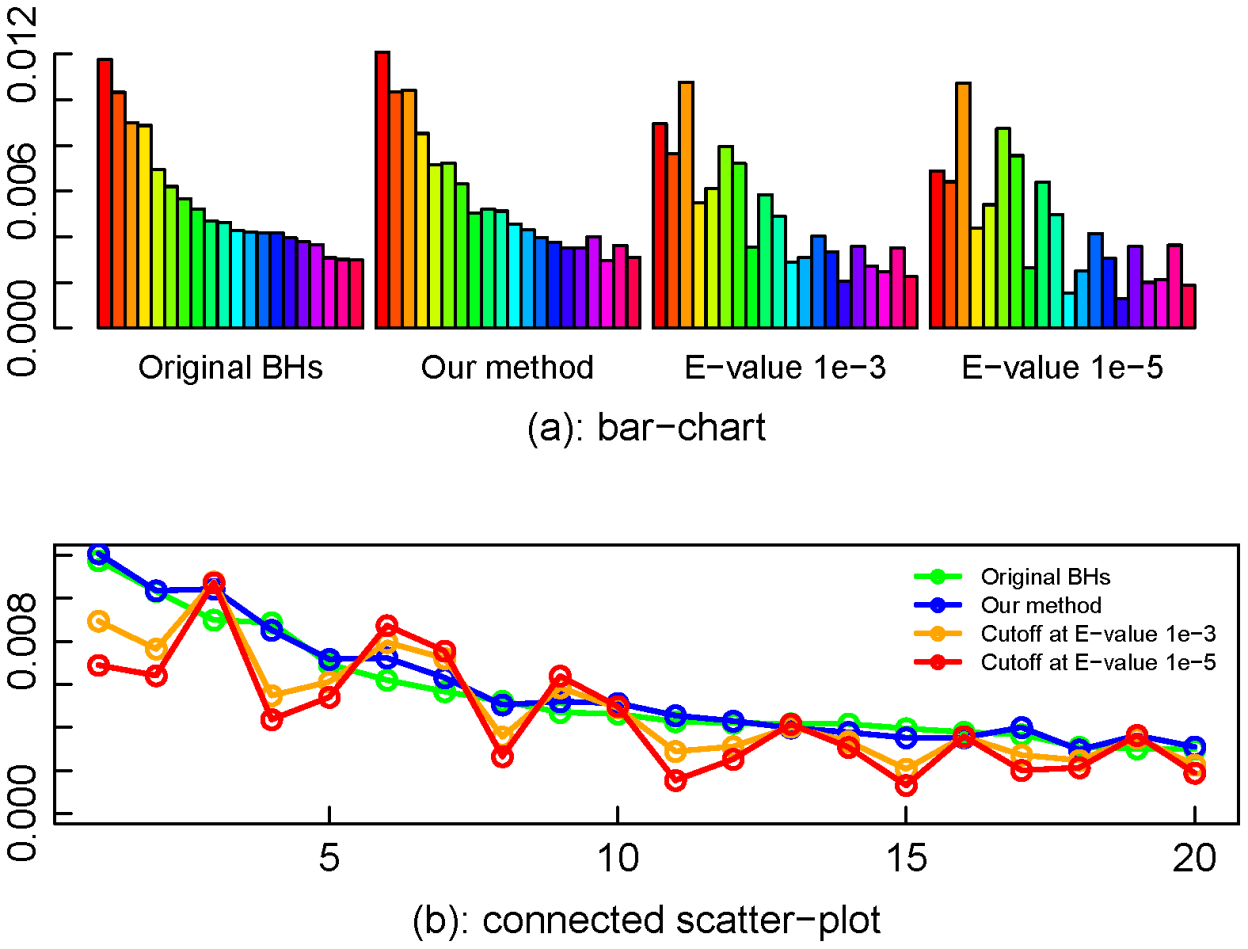
Partial functional profiles for M3_1X by different methods. The plots show that for the data set with coverage greater than 1, the proposed method outperforms the E-value cutoff methods in keeping the fidelity of the functional profile.

The comparison of the partial functional profiles by different methods is presented in **Figure 8** and again demonstrates the best performance by our proposed method in keeping the fidelity of the functional profile.

### Result from the data set with read coverage greater than 1

For the M3_2X dataset, the BLAST search detected 4546 COGs (3142 true COGs and 1404 artificial COGs). After score filtration, there were 985 (70%) artificial COGs removed, while 3043 true COGs were successfully preserved and 99 (3%) true COGs were erroneously filtered out. On average, only 5 reads were assigned to each of these lost true COGs (the largest read counts in this group was 26). Again, we regard these COGs as trivial entries in the construction of the functional profiles compared to the preserved COGs. Additional influential artificial COGs were detected by **Steps 1 & 2** and listed in **Table 8**. The values of RRC for the COGs identified in **Step 1** were 0, 0, 0.018 and 0.038, respectively. Comparison of partial functional profiles by the different methods (figure not shown) has the same conclusion as those in the previous two subsections.

**Table 8:** Influential artificial COGs in M3_2X detected by Step 1 and Step 2.

	COGs	Estimated No. of truly existing CDSs	Read Counts	
			By BLAST	By RPS-BLAST
Step 1	COG0454	0	813	0
	COG0500	4	1338	0
	COG0582	36	713	13
	COG0477	0	4703	177
Step 2	COG0457	0	226	0

Note: Two columns of read counts are obtained before score filtration (Step 1) and after score filtration (Step 2).

Combining the results from the three data sets, we observe that the percentage of artificial COGs by BLAST is positively related to the read coverage. This is a serious problem because the higher coverage means better assembly and becomes practically possible as the cost of sequencing is dropping. The proposed method successfully filtered out most (>70%) of the artificial COGs, though the percentage of artificial COGs removed decreases as the read coverage increases. Interestingly, the percentage of true COGs erroneously removed drops simultaneously.

## Discussion

Next-generation sequencing technology is dramatically changing the scenery in genomic/proteomic studies. The technology for functional metagenomic profiling of microbiota has been widely applied in environmental, biological and medical research. While the BLAST homolog searching tool is powerful in detecting COG families, it also introduces a significant amount of artificial functional families to the data. Successfully filtering out these families is essential to the construction of functional profile and the down-stream analysis. Current methods in common practice include E-value cutoffs, such as 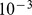 or 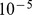. As demonstrated in previous sections, choosing the homologues with any of these E-value cutoffs alone decreases the sensitivity of true COGs, and also affects the proportional abundance of a COG in the profile.

Motivated by the empirical distributions of similarity scores of true (false) short homologies in the simulated data, we applied in this work the normalized penalty minimization method to obtain an efficient similarity score cutoff of 66 for metagenome with short reads of length ∼100 nt. By this rule, we could successfully filter out more than 70% of the artificial COGs generated by BLAST. Though it is inevitable that the filtering will result in the loss of some true COG families, we find that each of these lost true COGs has, on average, low read count and thus is regarded as trivial entries in the construction of the functional profile. By incorporating the RPS-BLAST searching results into the BLAST outputs, we propose to use Quadratic Discriminant Analysis (**Step 1**) and **Step 2** to further filter out the artificial COG families that cannot be removed by score filtration and have large read counts (i.e., influential artificial COGs).

Read coverage is an important factor in metagenomic studies. It affects the assembly accuracy and integrity [27]. By analyzing three experimental metagenomic data sets that consist of the same genomes, but have different read coverages, we showed that the proposed method can filter out most of artificial and influential artificial COGs in these experiments, and thus, is robust against the read coverage.

Another important issue in metagenomic functional profiling is the across annotations of COGs [6]. This can greatly lower the accuracy of the functional profile and needs to be corrected. Developing methods for the correction will be one of our future research topics.

## Supporting Information

Text S1 Steps to show that, for the simulated metagenome, we are highly confident that the score cutoff value should be between 63 and 68 in order to achieve a sensitivity of about 0.75.
(DOCX)
