# Supplementary material for: An Artificial Functional Family Filter in Homolog Searching in Next-generation Sequencing Metagenomics
Source: PLoS One. 2013 Mar 14;8(3):e58669. doi: 10.1371/journal.pone.0058669 (PMC3597637; doi:10.1371/journal.pone.0058669)
Supplement: Text S1 — Steps to show that, for the simulated metagenome, we are highly confident that the score cutoff value should be between 63 and 68 in order to achieve a sensitivity of about 0.75. (DOCX) [file pone.0058669.s001.docx]

**Text S1.** **Steps to show that, for the simulated metagenome, we are highly confident that the score cutoff value should be between 63 and 68 in order to achieve a sensitivity of about 0.75.**

Let$X$denote the variable of similarity score of TSH generated from a genome, which is a positive integer ranging approximately from 40 to 250. For any$p\in(0, 1)$, the population *p* percentile is defined as any value $\theta_{p}$for which$P(X\leq\theta_{p})\geq p$and$P(X\geq\theta{}_{p})\geq1-p$. By this definition, if a score cutoff value is chosen at $\theta_{p}$, the sensitivity achieved is at least $1-p$. Given a cutoff, the estimate of this sensitivity can be chosen as the percentage of TSHs with similarity scores being greater than the cutoff value (the true positive rate).

Let$X_{1},X_{2}\cdots X_{n}$be a sample of similarity scores of TSHs generated from a genome. A distribution-free confidence interval of $\theta_{p}$can be calculated as,

$$P(X_{(r)}\leq\theta_{p}\leq X_{(s)})\geq\sum_{i=r}^{s-1} \left(\begin{matrix} n \\ i \end{matrix}\right)p^{i}(1-p)^{n-i}$$

where$X_{(j)}$denotes the $j$th ordered distinct values of $X_{1},X_{2}\cdots X_{n}$, and $1\leq r<s\leq n$. **Table A** gives, for each genome, the probability that $\theta_{0.25}$falls in the interval $[63, 68]$. These probabilities are all greater than 0.99, and 8 of them are almost 1.

**Table A.** The probability of $63\leq\theta_{0.25}\leq68$

| Genome | $P(63\leq\theta_{0.25}\leq68)\geq$ |
| --- | --- |
| NC_007778 | 1 |
| NC_008255 | 0.9999 |
| NC_007644 | 1 |
| NC_007354 | 0.9947 |
| NC_007404 | 1 |
| NC_007335 | 1 |
| NC_007925 | 1 |
| NC_007947 | 1 |
| NC_007406 | 1 |
| NC_007958 | 1 |

Let$M$denote the variable of mixture of similarity scores of TSH, $S_{1}$and$S_{2}$, generated from two different genomes respectively.

And,

$P(M=S_{1})=\tau$ and$P(M=S_{2})=1-\tau$,

where the value of$\tau$is the composition proportion of similarity scores of TSH from genome 1 and $1-\tau$ is that from genome 2.

Let $\theta_{p}^{1}$ be the$p$percentile of similarity scores of TSH from genome 1; $\theta_{p}^{2}$from genome 2.

Case 1: $\theta_{p}^{1}=\theta_{p}^{2}$. Choose$\theta=\theta_{p}^{1}=\theta_{p}^{2}$, then

$$P(M\leq\theta)=P(M\leq\theta_{p}^{1}|M=S_{1})P(M=S_{1})+P(M\leq\theta_{p}^{2}|M=S_{2})P(M=S_{2})$$

$$=P(S_{1}\leq\theta_{p}^{1})P(M=S_{1})+P(S_{2}\leq\theta_{p}^{2})P(M=S_{2})$$

$$\geq p\tau+p(1-\tau)=p;$$

$$P(M\geq\theta)=P(M\geq\theta_{p}^{1}|M=S_{1})P(M=S_{1})+P(M\geq\theta_{p}^{2}|M=S_{2})P(M=S_{2})$$

$$=P(S_{1}\geq\theta_{p}^{1})P(M=S_{1})+P(S_{2}\geq\theta_{p}^{2})P(M=S_{2})$$

$$\geq(1-p)\tau+(1-p)(1-\tau)=1-p;$$

because the distribution of similarity scores of TSHs generated from a genome is independent to how the genome mixed with the other one. We showed $\theta$ is $\theta_{p}$, the $p$percentile of *M*.

Case 2: $\theta_{p}^{1}<\theta_{p}^{2}$.

We assume that there is at least one similarity score, either from $S_{1}$or$S_{2}$, in the interval $[\theta_{p}^{1}, \theta_{p}^{2}]$. Otherwise, it is easy to show that any value between $\theta_{p}^{1}$ and $\theta_{p}^{2}$can be the $p$percentile of *M.*

$$P(M\leq\theta_{p}^{2})=P(M\leq\theta_{p}^{2}|M=S_{1})P(M=S_{1})+P(M\leq\theta_{p}^{2}|M=S_{2})P(M=S_{2})$$

$$=P(S_{1}\leq\theta_{p}^{2})P(M=S_{1})+P(S_{2}\leq\theta_{p}^{2})P(M=S_{2})$$

$$>P(S_{1}\leq\theta_{p}^{1})P(M=S_{1})+P(S_{2}\leq\theta_{p}^{2})P(M=S_{2})$$

$$=p\tau+p(1-\tau)=p;$$

$$P(M\geq\theta_{p}^{1})=P(M\geq\theta_{p}^{1}|M=S_{1})P(M=S_{1})+P(M\geq\theta_{p}^{1}|M=S_{2})P(M=S_{2})$$

$$=P(S_{1}\geq\theta_{p}^{1})P(M=S_{1})+P(S_{2}\geq\theta_{p}^{1})P(M=S_{2})$$

$$>P(S_{1}\geq\theta_{p}^{1})P(M=S_{1})+P(S_{2}\geq\theta_{p}^{2})P(M=S_{2})$$

$$=(1-p)\tau+(1-p)(1-\tau)=1-p;$$

Thus, under Case 2, the $p$percentile of *M* is between $[\theta_{p}^{1}, \theta_{p}^{2}]$, or $\theta_{p}^{1}\leq\theta{}_{p}\leq\theta_{p}^{2}$.

Case 3: $\theta_{p}^{1}>\theta_{p}^{2}$. Similarly we can have $\theta_{p}^{2}\leq\theta{}_{p}\leq\theta_{p}^{1}$.

Following these results, if $\theta_{p}^{1}$ and $\theta_{p}^{2}$ both bounded in the same region $[\theta_{L}, \theta_{U}]$, the $p$percentile of *M* is also bounded in $[\theta_{L}, \theta_{U}]$. That is,

$$\begin{matrix} \theta_{L}\leq\theta_{p}^{1}\leq\theta_{U} \\ \theta_{L}\leq\theta_{p}^{2}\leq\theta_{U} \\ \min(\theta_{p}^{1}, \theta_{p}^{2})\leq\theta_{p}\leq\max(\theta_{p}^{1}, \theta_{p}^{2}) \end{matrix}\Rightarrow\theta_{L}\leq\theta_{p}\leq\theta_{U}$$

We considered only the mixture of two genomes. However, the proof can be generalized to the mixture of more than two genomes. Thus, for the case of $n$genomes,

$$P(\theta_{L}\leq\theta_{p}\leq\theta_{U})\geq P(\theta_{L}\leq\theta_{p}^{1}\leq\theta_{U},\cdots,\theta_{L}\leq\theta_{p}^{n}\leq\theta_{U})$$

$$\geq1-\sum_{i=1}^{n} P(\theta_{p}^{i}>\theta_{U}, \theta_{p}^{i}<\theta_{L})$$

$$=1-\sum_{i=1}^{n} \{1-P(\theta_{L}\leq\theta_{p}^{i}\leq\theta_{U})\}$$

For the simulated ~100nt metagenome, the probability of $\theta_{0.25}$ falling in [63, 68] is

$P(63\leq\theta_{0.25}\leq68)\geq1-(1-0.9999)-(1-0.9947)=0.9946$.
